# *In vitro* activity of human defensins HNP-1 and hBD-3 against multidrug-resistant ESKAPE Gram-negatives of clinical origin and selected peptidoglycan recycling-defective mutants

**DOI:** 10.1128/spectrum.00358-24

**Published:** 2024-03-05

**Authors:** María Escobar-Salom, Isabel María Barceló, Estrella Rojo-Molinero, Elena Jordana-Lluch, Gabriel Cabot, Antonio Oliver, Carlos Juan

**Affiliations:** 1 Research Unit, University Hospital Son Espases-Health Research Institute of the Balearic Islands (IdISBa), Palma, Spain; 2 Microbiology Department, University Hospital Son Espases, Palma, Spain; 3 Centro de Investigación Biomédica en Red de Enfermedades Infecciosas (CIBERINFEC), Madrid, Spain; JMI Laboratories, North Liberty, Iowa, USA

**Keywords:** ESKAPE Gram-negatives, *Pseudomonas aeruginosa*, *Klebsiella pneumoniae*, *Enterobacter cloacae *complex, *Acinetobacter baumannii*, Human Neutrophil Peptide-1 (HNP-1), human Beta Defensin 3 (hBD-3), peptidoglycan recycling

## Abstract

The use of immune compounds as antimicrobial adjuvants is a classic idea recovering timeliness in the current antibiotic resistance scenario. However, the activity of certain antimicrobial peptides against ESKAPE Gram-negatives has not been sufficiently investigated. The objective of this study was to determine the activities of human defensins HNP-1 and hBD-3 alone or combined with permeabilizing/peptidoglycan-targeting agents against clinical ESKAPE Gram-negatives [*Acinetobacter baumannii* (AB), *Enterobacter cloacae* (EC), *Klebsiella pneumoniae* (KP), and acute/chronic *Pseudomonas aeruginosa* (PA)]. Lethal concentrations (LCs) of HNP-1 and hBD-3 were determined in four collections of multidrug resistant EC, AB, KP, and PA clinical strains (10–36 isolates depending on the collection). These defensins act through membrane permeabilization plus peptidoglycan building blockade, enabling that alterations in peptidoglycan recycling may increase their activity, which is why different recycling-defective mutants were also included. Combinations with physiological lysozyme and subinhibitory colistin for bactericidal activities determination, and with meropenem for minimum inhibitory concentrations (MICs), were also assessed. HNP-1 showed undetectable activity (LC > 32 mg/L for all strains). hBD-3 showed appreciable activities: LC ranges 2–16, 8–8, 8->32, and 8->32 mg/L for AB, EC, KP, and PA, being PA strains from cystic fibrosis significantly more resistant than acute origin ones. None of the peptidoglycan recycling-defective mutants showed greater susceptibility to HNP-1/hBD-3. Combination with colistin or lysozyme did not change their bactericidal power, and virtually neither did meropenem + hBD-3 compared to meropenem MICs. This is the first study comparatively analyzing the HNP-1/hBD-3 activities against the ESKAPE Gram-negatives, and demonstrates interesting bactericidal capacities of hBD-3 mostly against AB and EC.

**IMPORTANCE:**

In the current scenario of critical need for new antimicrobials against multidrug-resistant bacteria, all options must be considered, including classic ideas such as the use of purified immune compounds. However, information regarding the activity of certain human defensins against ESKAPE Gram-negatives was incomplete. This is the first study comparatively assessing the in vitro activity of two membrane-permeabilizing/peptidoglycan construction-blocking defensins (HNP-1 and hBD-3) against relevant clinical collections of ESKAPE Gram-negatives, alone or in combination with permeabilizers, additional peptidoglycan-targeting attacks, or the blockade of its recycling. Our data suggest that hBD-3 has a notable bactericidal activity against multidrug-resistant *Acinetobacter baumannii* and *Enterobacter cloacae* strains that should be considered as potential adjuvant option. Our results suggest for the first time an increased resistance of *Pseudomonas aeruginosa* strains from chronic infection compared to acute origin ones, and provide new clues about the predominant mode of action of hBD-3 against Gram-negatives (permeabilization rather than peptidoglycan-targeting).

## INTRODUCTION

The global increase in antibiotic resistance levels mostly in certain species such as ESKAPE pathogens—standing out because of the morbidity-mortality of their infections and associated economic burden—makes the continuous quest for therapeutic alternatives essential (1). Although some new drugs (e.g., β-lactam-β-lactamase inhibitor combinations and cefiderocol) showing strong activity against ESKAPE Gram-negatives (*Klebsiella pneumoniae*, *Acinetobacter baumannii*, *Pseudomonas aeruginosa*, and *Enterobacter* spp.) have recently been introduced in clinical practice, different reports of resistance to them are progressively appearing (2, 3); thus, the pipeline of new therapeutic strategies must continue operating. The exogenous administration of purified immune compounds as antimicrobial adjuvants is a classic idea (4) that could recover timeliness in this scenario, but data on the *in vitro* activity of certain antimicrobial peptides against ESKAPE Gram-negatives, or at least some of their representatives, are surprisingly scarce (4). This is the case of two defensins, namely HNP-1 (Human Neutrophil Peptide-1, also known as human alpha defensin-1) and hBD-3 (human Beta-Defensin-3), which act, at least partially, like most of the rest of cationic antimicrobial peptides: through membrane permeation and derived osmotic lysis (4, 5). What makes HNP-1 and hBD-3 particularly interesting is their double mode of action, since they also bind the peptidoglycan precursor lipid II, blocking the addition of new material into the cell wall. Lipid II, carrying the peptidoglycan monomers (N-acetyl-glucosamine-N-acetyl-muramic acid-pentapeptides), is normally translocated across the cytosolic membrane enabling their incorporation into peptidoglycan through the transglycosylase activity of certain penicillin-binding proteins (PBPs) (4, 5). Yet in the presence of the aforementioned defensins, lipid II blockade indirectly weakens the cell-wall structure by disabling the incorporation of new material and leading to cell lysis, a phenomenon somehow similar to the mode of action of β-lactams. This mechanism was thought to be significant only against Gram-positives since access to lipid II is theoretically easier due to the absence of an outer membrane (6–8). Nevertheless, although the affinity of HNP-1 and hBD-3 toward lipid II is apparently lower than other elements targeting this precursor, these defensins also display a significant activity against Gram-negatives not shown by other lipid II-binding resources (4, 6–8). However, the exact predominant mechanism through which HNP-1/hBD-3 display anti-Gram-negative activity (permeabilization alone leading to direct lysis and/or lipid II blockade), remains elusive. Moreover, few studies have analyzed the real activity of these two defensins against Gram-negatives *in vitro*. This is the case of some that exclusively used reference strains of *P. aeruginosa* and in fewer cases of *K. pneumoniae* or *A. baumannii* (9–15). In addition, the analysis of HNP-1/hBD-3 activity against clinical strains has barely been addressed, with a limited number of *K. pneumoniae* strains or a very low number of *A. baumannii* and *P. aeruginosa* isolates (16, 17). Finally, no single study has assessed HNP-1 or hBD-3 activity on clinical *Enterobacter cloacae* complex strains, altogether posing knowledge gaps that we sought to fill here. Thus, this is a pioneer comparative study assessing lipid II-targeting defensin activities against representatives of all the ESKAPE Gram-negatives in parallel, clearly upgrading the previous knowledge on the topic.

Interest in HNP-1 and hBD-3 as therapeutic allies is supported by studies showing that their malfunction, as happens in contexts such as cystic fibrosis, asthma, and so on, triggers an enhanced susceptibility to infection (4, 18–20). This fact suggests the excellent protective activity of HNP-1 and hBD-3 in regular conditions and their consequent exploitable antimicrobial potential. In addition, we hypothesize that if parallel peptidoglycan damages were added to the membrane permeabilization/blockade of lipid II (both activities allegedly exerted by HNP-1/hBD-3), more catastrophic consequences would be obtained for bacterial viability. This idea resembles those described in *P. aeruginosa* in which (i) outer membrane permeabilization drastically enhances susceptibility to peptidoglycan-lytic attacks (lysozyme and peptidoglycan-recognition proteins, PGLYRPs); and (ii) mutants with altered peptidoglycan recycling become more vulnerable to these aggressions (21, 22). However, the possibilities of peptidoglycan recycling blockade being a strategy to sensitize bacteria against lipid II-targeting defensins, and increasing their power through the parallel administration of permeabilizers/peptidoglycan-lytic agents, have not been investigated. Thus, in order to enrich knowledge in the field, we analyzed the antibacterial activities of HNP-1 and hBD-3 in different collections of clinical ESKAPE multi-drug resistant Gram-negatives and in a set of peptidoglycan-recycling defective mutants, together with the effect of the combinations with other peptidoglycan-targeting attacks (physiological concentrations of lysozyme and the relevant β-lactam meropenem) or additional permeabilizers (subinhibitory colistin).

## RESULTS

HNP-1 showed virtually no detectable activity against the studied strains, with LCs > 32 mg/L (>9.3 µM) in all cases except for the *M. lysodeikticus* control (Tables 1 and 2). Even MBC values were also >32 mg/L in most of the strains, with the exception of the whole set of *A. baumannii* isolates and the *M. lysodeikticus* control (32 and 8 mg/L, respectively). In light of this unexpectedly low level of activity, assays were repeated dissolving the peptide in 0.01% acetic acid as previously described (11), yet no changes in LCs/MBCs were seen (data not shown).

**TABLE 1:** *In vitro* activity of HNP-1 and hBD-3 against the *P. aeruginosa* strains studied in this work, whose relevant features are also shown

*S*trains	PFGE clone	ST	Resistance profile^*a*^	LC (mg/L) HNP-1	MBC (mg/L) HNP-1	LC (mg/L) hBD-3	MBC (mg/L) hBD-3
*Micrococcus lysodeikticus* ATCC 4698	−	ND	ND	16	8	8	4
*P. aeruginosa* reference strains/mutants							
PAO1	−	549	MultiS	>32	>32	8	4
PAO1 ΔAmpG	−	549	MultiS	>32	>32	8	4
PAO1 ΔNagZ	−	549	MultiS	>32	>32	16	4
PAO1 ΔADADh2ADh3	−	549	ModR	>32	>32	16	4
PA14	−	253	MultiS	>32	>32	8	4
PA14 ΔAmpG	−	253	MultiS	>32	>32	8	4
PA14 ΔNagZ	−	253	MultiS	>32	>32	8	4
PA14 ΔADADh2ADh3	−	253	ModR	>32	>32	8	4
Bloodstream infection-proceeding *P. aeruginosa* strains							
PABA-12	1	175	XDR	>32	>32	16	8
PABA-19	3	111	MDR	>32	>32	16	8
PABA-28	11	244	ModR	>32	>32	8	4
PABA-37	26	699	ModR	>32	>32	8	4
PABA-43	2	175	XDR	>32	>32	16	4
PABA-48	106	319	ModR	>32	>32	16	4
PABA-63	22	446	MDR	>32	>32	16	8
PABA-100	30	1,113	MultiS	>32	>32	16	8
PABA-120	31	1,114	ModR	>32	>32	8	4
PABA-142	35	1,178	MultiS	>32	>32	8	4
PABA-181	ND	385	ModR	>32	>32	16	4
PABA-211	3	111	MDR	>32	>32	16	8
Cystic fibrosis infection-proceeding *P. aeruginosa* strains^*b*^							
PAFQ05-*03*	E	1,108	MDR	>32	>32	16	8
PAFQ05-*11*	E	1,108	MDR	>32	>32	16	4
PAFQ06-*03*	A	274	MultiS	>32	>32	>32	32
PAFQ06-*10*	A	274	ModR	>32	>32	>32	32
PAFQ10-*03*	A	274	ModR	>32	>32	16	4
PAFQ10-*11*	A	274	MDR	>32	>32	16	4
PAFQ11-*03*	K	701	MultiS	>32	>32	16	8
PAFQ11-*10*	K	701	MDR	>32	>32	>32	32
PAFQ12-146-*07*	B	146	XDR	>32	>32	16	8
PAFQ12-146-*10*	B	146	ModR	>32	>32	16	8
PAFQ12-299-*03*	C	299	ModR	>32	>32	>32	32
PAFQ12-299-*06*	C	299	MDR	>32	>32	>32	32
PAFQ15-*03*	A	274	MultiS	>32	>32	16	4
PAFQ15-*10*	A	274	ModR	>32	>32	8	4
PAFQ16-*03*	M	1,073	ModR	>32	>32	16	8
PAFQ16-*10*	M	1,073	MDR	>32	>32	>32	32
PAFQ21-1088-*03*	H	1,088	MultiS	>32	>32	8	8
PAFQ21-1088-*10*	H	1,088	ModR	>32	>32	8	8
PAFQ21-1109-*05*	I	1,109	XDR	>32	>32	8	4
PAFQ21-1109-*10*	I	1,109	MDR	>32	>32	8	4
PAFQ24-*04*	A	1,089	MDR	>32	>32	16	8
PAFQ24-*10*	A	1,089	ModR	>32	>32	16	16
PAFQ28-*06*	J	1,071	ModR	>32	>32	>32	16
PAFQ28-*10*	J	1,071	ModR	>32	>32	16	16

^*a*^ Resistance profiles [multidrug-resistant (MDR), extensively drug-resistant (XDR)] were assigned following previously established criteria (23). Moreover, MultiS (multi-susceptible) indicates susceptibility to all categories of antibiotics, while ModR (moderately resistant) indicates resistance to one or two categories (24).

^*b*^ Pairs of sequential isogenic isolates [early-late, obtained with a difference of at least 3 years (italicized numbers indicate year of isolation) from a total of 10 patients (thus, from 2 of the patients, 2 pairs of isolates were obtained on each, at different time points)] (25); Other abbreviations: LC: lethal concentration; MBC: minimum bactericidal concentration; PFGE: Pulsed Field Gel Electrophoresis; ST: sequence type; ND: not determined.

**TABLE 2:** *In vitro* activity of HNP-1 and hBD-3 against the *A. baumanii, E. cloacae* complex, and *K. pneumoniae* strains studied in this work, whose relevant features are also shown

Species/strain	PFGE clone	ST	Acquired β-lactamases	Resistance profile^*a*^	LC (mg/L) HNP-1	MBC (mg/L) HNP-1	LC (mgL) hBD-3	MBC (mg/L) hBD-3
*A. baumannii*								
ATCC 19606	ND	52	−	MultiS	>32	32	4	1
AB1	ND	2	OXA-23	XDR	>32	32	4	1
AB2	ND	2	OXA-23	XDR	>32	32	4	1
AB3	ND	2	OXA-23	XDR	>32	32	4	1
AB4	ND	2	OXA-23	XDR	>32	32	8	1
AB5	ND	2	OXA-23	XDR	>32	32	8	1
AB6	ND	2	OXA-23	XDR	>32	32	2	1
AB7	ND	2	OXA-23	XDR	>32	32	4	1
AB8	ND	2	OXA-23	MDR	>32	32	2	1
AB9	ND	2	OXA-23	XDR	>32	32	16	8
AB10	ND	2	OXA-23	XDR	>32	32	2	1
*E. cloacae* complex								
ATCC 13047	ND	1	−	ModR	>32	>32	8	4
ATCC 13047 ΔAmpG	ND	1	−	ModR	>32	>32	8	4
EC1	ECLO1	78	VIM-1, OXA-1::IS	MDR	>32	>32	8	4
EC2	ECLO2	133	VIM-1, CTX-M-9, OXA-1::IS	MDR	>32	>32	8	4
EC3	ECLO3	1074	VIM-1, OXA-1, CTX-M-9	XDR	>32	>32	8	4
EC4	ECLO4	1074	VIM-1, OXA-1	MDR	>32	>32	8	4
EC5	ECLO6	108	VIM-1, OXA-1	MDR	>32	>32	8	4
EC6	ECLO5	93	VIM-1, CTX-M-9, OXA-1::IS	MDR	>32	>32	8	8
EC7	ECLO7	110	VIM-1, OXA-1, SHV-12	MDR	>32	>32	8	4
EC8	ECLO8	175	VIM-1, CTX-M-9, OXA-1::IS	XDR	>32	>32	8	4
EC9	ECLO10	114	VIM-1, CTX-M-9, OXA-1::IS	XDR	>32	>32	8	4
EC10	ECLO9	145	VIM-1, CTX-M-9, OXA-1::IS	MDR	>32	>32	8	4
*K. pneumoniae*								
Kp52.145	ND	66	−	MultiS	>32	>32	16	4
Kp52.145 ΔAmpG	ND	66	−	MultiS	>32	>32	8	4
ATCC 700721	ND	38	SHV-11, SHV-12, TEM-1, OXA-9	MDR	>32	>32	16	8
ATCC 700721 ΔAmpG	ND	38	SHV-11, SHV-12, TEM-1, OXA-9	MDR	>32	>32	16	8
KP1	K1	11	VIM-1, OXA-1, DHA-1	XDR	>32	>32	8	4
KP2	K5	11	VIM-1	XDR	>32	>32	8	4
KP3	K2	17	OXA-48, TEM-1B, CTX-M-15	XDR	>32	>32	16	4
KP4	K3	556	VIM-1, TEM-201, OXA-1::IS, CTX-M-9	XDR	>32	>32	>32	32
KP5	K4	36	VIM-1, OXA-1, CTX-M-14, CMY-2	XDR	>32	>32	8	4
KP6	K6	321	VIM-1	XDR	>32	>32	16	8
KP7	K7	392	VIM-1, CTX-M-9, CTX-M-15, TEM-1B, OXA-1	MDR	>32	>32	>32	>32
KP8	K8	461	VIM-1, OXA-1, DHA-1	XDR	>32	>32	8	4
KP9	K9	290	OXA-9, NDM-1, CTX-M-15, TEM-1B	XDR	>32	>32	16	4
KP10	K12	98	VIM-1, OXA-1	XDR	>32	>32	8	4
KP11	K1	11	VIM-1, CTX-M-9	XDR	>32	>32	>32	>32
KP12	ND	512	KPC-3	XDR	>32	>32	>32	>32

^*a*^ Resistance profiles [multidrug-resistant (MDR), extensively drug-resistant (XDR)] were assigned following previously established criteria (23). Moreover, MultiS (multi-susceptible) indicates susceptibility to all categories of antibiotics, while ModR (moderately resistant) indicates resistance to one or two categories (24). Other abbreviations: LC: lethal concentration; MBC: minimum bactericidal concentration; PFGE: Pulsed Field Gel Electrophoresis; ST: sequence type; ND: not determined; IS: insertion sequence.

On the other hand, hBD-3 showed appreciable activities in the majority of strains regardless of most of their characteristics. In other words, there were no clear trends in terms of increased/reduced susceptibility to this defensin depending on clonal lineage (high-risk clones vs not), harbored β-lactamases, or antibiotic resistance profile. In this last regard, since polymyxin resistance mechanisms have been related to cross-resistance to different antimicrobial peptides (4), clinical strains displaying colistin resistance could have been expected to show significantly decreased susceptibility to hBD-3. However, KP11, KP12, PAFQ12-146-07, PAFQ21-1088-10, and PABA-48 strains—standing out because of their colistin resistance profile (respective MICs of 48, 4, 8, 4, and 8 mg/L)—did not show a particularly resistant phenotype against hBD-3 compared to other strains devoid of colistin resistance but with equal or even higher LC values (Tables 1 and 2).

Returning to hBD-3-related general data (Tables 1 and 2), as an indicator of the overall activity of this defensin, the lowest LC values for at least 90% of the clinical isolates of each species were 8 mg/L for both *A. baumannii* and *E. cloacae* complex and >32 mg/L for both *K. pneumoniae* and *P. aeruginosa*. Likewise, the lowest LC values for at least 50% of the corresponding clinical isolates were 4 mg/L for *A. baumannii*, 8 mg/L for *E. cloacae* complex, and 16 mg/L for both *K. pneumoniae* and *P. aeruginosa*. The respective LC ranges were: 2–16, 8–8, 8->32, and 8->32 mg/L (values in µM: 0.38–3.1, 1.55–1.55, 1.55->6.2, and 1.55->6.2). Therefore, the activity of hBD-3 was apparently greater against *A. baumannii* followed by *E. cloacae* complex, with several strains of the former with LCs even below that of the Gram-positive control (15). Moreover, as could be expected, MBC values were overall lower than those of LCs: generally 1/2 or 1/4 of the latter. Nevertheless, in some specific cases, there were no changes between LC and MBC values (PAFQ21-1088-*03*, PAFQ21-1088-*10*, PAFQ24-*10*, PAFQ28-*10*, and EC6 strains). In others, the potential variations were not measurable because LC and MBC values were both >32 mg/L (KP7, KP11, and KP12) (Tables 1 and 2).

Interestingly, *P. aeruginosa* strains from cystic fibrosis were seemingly more resistant to hBD-3 than those from bloodstream infection. In this regard, the lowest MBC for at least 90% of the bloodstream isolates was 8 mg/L, whereas that parameter in cystic fibrosis-proceeding strains was 32 mg/L (both considering the whole cystic fibrosis collection or splitting it into early vs late isolates). Moreover, a clear trend of increased resistance to hBD-3 of late cystic fibrosis isolates (MBC: 32 mg/L) compared to early ones (MBC: 8 mg/L) was seen in two pairs of isolates: PAFQ11 and PAFQ16 (Table 1).

None of the peptidoglycan recycling-defective mutants showed greater susceptibility to the tested defensins than the parental strains , whose LC and MBC values were generally in the low range compared to the respective species’ clinical isolates (Tables 1 and 2). Since peptidoglycan recycling is a key pipeline for the generation of new material to be added to the cell wall, it is likely to be more important in situations in which bacteria divide or have to repair peptidoglycan damage (4, 22). For this reason, we hypothesized that the 2.5 h of incubation with defensins could be too short a period to see the potentially deleterious effects of the peptidoglycan recycling blockade during a lipid II-targeting attack. Consequently, the hBD-3 assays were repeated with PAΔAG, PAΔnZ, PAΔDDh2Dh3, Kp52.145RΔAG, MGH 78578ΔAG, and ATCC 13047ΔAG mutants and the respective wild-type strains, whilst prolonging the incubation period to 18 h. In these conditions, recycling would be expected to play a more important role in enabling minimum growth/repair of peptidoglycan damage, which should be disabled after lipid II blockade by defensins. Nevertheless, LCs did not change in comparison to the 2.5 h incubation results (data not shown).

Regarding the different studied combinations, those of hBD-3 with subinhibitory colistin or human lysozyme at physiological concentration did not change LC values in the selected strains (Table 3), and the same happened for HNP-1 (all LCs for the combined treatments were >32 mg/L, data not shown). MBC values for these hBD-3 combined treatments followed the same trend, although seemingly, in the specific case of PAO1 MBCs, there was a slight increase in activity after combination with lysozyme (decrease of one dilution when comparing hBD-3 + lysozyme with hBD-3 alone) (Table 3).

**TABLE 3:** *In vitro* activity of hBD-3 alone or combined with the indicated agents against selected *P. aeruginosa*, *K. pneumoniae, E. cloacae* complex, and *A. baumannii* strains

Treatment added to hBD-3	Species/strains							
	***P. aeruginosa*** PAO1		***K. pneumoniae*** ATCC 700721		***E. cloacae*** ATCC 13047		***A. baumannii*** ATCC 19606	
	LC (mg/L)	MBC (mgL)	LC (mg/L)	MBC (mg/L)	LC (mg/L)	MBC (mg/L)	LC (mg/L)	MBC (mg/L)
None	8	4	16	8	8	4	4	1
Human lysozyme 25 mg/L	8	2	16	8	8	4	ND	ND
Colistin^*a*^ 0.025 mg/L	8	4	16	8	8	4	4	1

^*a*^ Colistin minimum inhibitory concentrations determined through E-test were 0.25 mg/Lfor *E. cloacae* and *K. pneumoniae*, and 1 mg/L for the *A. baumannii* and *P. aeruginosa* reference strains used. Other abbreviations: LC: lethal concentration; MBC: minimum bactericidal concentration. ND: not determined, because the used *A. baumannii* strain was extremely susceptible to the physiological concentration of lysozyme *per se*, i.e., >99% of killing. The control treatments (lysozyme alone or colistin alone in the indicated concentrations) never caused a bacterial killing above 50% of the initial inoculum in the rest of species/strains (data not shown).

Although hBD-3 has been shown to display a limited growth inhibition capacity *per se* in typical broth microdilution assays against several Gram-negatives (15, 26), we hypothesized that this antimicrobial peptide could still work as an adjuvant for other peptidoglycan-targeting agents such as β-lactams. In this vein, we sought to investigate whether some of the alleged mechanisms of this defensin (membrane permeation and/or peptidoglycan building blockade) could increase the antibacterial effects of these antibiotics, specifically carbapenems because of their relevance and the growing resistance rates to them (1). For this reason, regular microdilution assays were performed to determine MICs of a representative carbapenem widely used against resistant ESKAPE pathogens, namely meropenem, either alone or in combination with the aforementioned defensin added at sub-inhibitory/sub-lethal concentrations. As can be seen in Table 4, hBD-3 alone displayed no measurable inhibition capacity against *P. aeruginosa*, *K. pneumoniae*, and *E. cloacae* strains (MICs always >32 mg/L, including the respective peptidoglycan recycling-defective mutants), albeit its power was higher against *A. baumannii* (MICs of 4 and 16 mg/L in the two studied strains) as previously reported (15). When added at sub-inhibitory/sub-lethal concentrations, this permeabilizing/lipid II-targeting agent had almost no effect increasing meropenem effectiveness against the strains tested (either carbapenem susceptible or resistant), although a minor decrease in specific *P. aeruginosa* and *K. pneumoniae* strains MICs (one dilution) was seen (Table 4).

**TABLE 4:** Minimum inhibitory concentrations (determined by broth microdilution) of hBD-3, meropenem, or combined treatment against *P. aeruginosa*, *K. pneumoniae, E. cloacae* complex, and *A. baumannii* reference strains, selected mutants, and clinical isolates

Species/strain	MIC (mg/L)		
	hBD-3	Meropenem	Meropenem + sub-lethal hBD-3^*a*^
*M. lysodeikticus* ATCC 4698	0.5	ND	ND
*P. aeruginosa*			
PAO1	>32	0.25	0.125
PAO1 ΔAmpG	>32	0.25	ND
PABA-12	>32	8	4
*K. pneumoniae*			
ATCC 700721	>32	0.032	0.016
ATCC 700721 ΔAmpG	>32	0.032	ND
KP12	>32	>32	>32
*E. cloacae*			
ATCC 13047	>32	0.125	0.125
ATCC 13047 ΔAmpG	>32	0.125	ND
EC9	>32	>32	>32
*A. baumannii*			
ATCC 19606	4	0.25	0.25
AB1	16	>32	>32

^*a*^ The concentrations of hBD-3 added in the combined treatments were always 1/4 of the respective LC values previously obtained for each strain (Table 1 and Table 2). ND: not determined.

## DISCUSSION

Human alpha defensin HNP-1 is predominantly present in the granules of polymorphonuclear cells and exerts its antimicrobial activity mostly in phagosomal or NETosis contexts (4). Meanwhile, hBD-3 is secreted by different epithelial cells (bronchi, oral and vaginal mucosa, tonsils, etc.) and also by keratinocytes or platelets. Although it undergoes a notable loss of activity when incubated in human serum because of different factors (16), its antibacterial power is more resistant to high salt concentrations than that of HNP-1 (11, 27–31). In this sense, the protective action of hBD-3 in infected wounds is well-known (32), as well as the essential role that its murine homolog plays in protection against corneal infection by *P. aeruginosa* (12), among other examples (4, 33). These circumstances demonstrate the excellent natural antibacterial activity of these defensins and their theoretical potential for being used as therapeutic adjuvants at least against infections happening in low salt contexts (e.g. mucosa, wounds, etc.). In this regard, injection of HNP-1 was shown to cause a significant (ca. 2 log units) leukocyte‐dependent *K. pneumoniae* burden reduction in the peritoneum and thigh in murine models, although probably at least partially owing to its immunomodulatory properties (34). Surprisingly, apart from this study *in vivo*, published information about the *in vitro* activity of HNP-1 or hBD-3 against ESKAPE Gram-negatives is scarce (mostly if referring to clinical strains) (4, 9–17). Our study with different clinically relevant collections of ESKAPE Gram-negatives poses an important complement to these previous partial results.

Regarding HNP-1, since the activity against our *M. lysodeikticus* control was lower than previously described (15), one could argue that the purchased peptide retained only a part of its original effectiveness. However, it must be considered that the *M. lysodeykticus* strain was not the same as in the mentioned study, which could explain the discrepancy (15). Moreover, when comparing the HNP-1 activity data from some other studies, our results do not seem to be that different. For instance, Takemura et al. found a lethal concentration >50 mg/L for an HNP-1/-2/-3 mixture against *P. aeruginosa* (9), whereas Campos et al. and Padilla et al. determined bactericidal concentrations of HNP-1 > 30 mg/L against *K. pneumoniae* (35, 36). Finally, data from Llobet et al. suggest bactericidal concentrations of HNP-1 that could be similar to ours, since they obtained MICs of around 10 mg/L against *K. pneumoniae* and *P. aeruginosa* (13). In light of all these data one could argue that HNP-1 may not have an important direct killing activity against Gram-negatives, and may exert its defensive function predominantly through other mechanisms *in vivo* (34), a question that in any case, our work did not aim to approach. It is true, however, that Varkey et al. and Zharkova et al. found a potent activity of HNP-1 against *P. aeruginosa* and *A. baumanii* type strains with LC/MIC values of 1/0.6 µM, respectively (11, 15), which would suggest a high intrinsic bactericidal power for this peptide. In any case, basing on our results with the collections and conditions we used, we conclude that HNP-1 cannot be envisaged as a promising bactericidal adjuvant for the moment. Future work with other purified HNP-1 and extended collections of strains should be performed to confirm this apparently poor bactericidal power against the studied species.

Dealing with hBD-3 bactericidal activity in our clinical/reference strains, our results are quite in accordance with data from several other studies, in which lethal or inhibitory concentrations (always using reference strains) between 10 and 100 mg/L for *P. aeruginosa* (12, 14, 26–31), ca. 19 mg/L for *K. pneumoniae* (14), ca. 7 mg/L for *E. cloacae* (10), and ca. 3–6 mg/L for *A. baumannii* (15, 37) were documented. It is also true, however, that another study (10) reported hBD-3 bactericidal concentrations up to ca. fivefold lower for some *P. aeruginosa* and *A. baumanii* ATCC strains compared to ours.

Much fewer studies on the activity of hBD-3 against clinical strains have been published. For instance, Maisetta et al. used a collection of only six *P. aeruginosa* and six *A. baumannii* isolates, obtaining results that are fairly comparable with ours, given the bactericidal concentrations they observed (between 4 and 8 mg/L). The study of Sahly et al. (17), with 36 clinical *K. pneumoniae* strains harboring different Extended-Spectrum β-lactamases, showed bactericidal concentrations in rather wide ranges (between 0.39 and 25 mg/L), which are overall lower than ours (8->32 mg/L). Regardless, the interest that our *K. pneumoniae* collection adds to these data is the well-characterized high resistance profile (all the strains harbored carbapenemases) and epidemiological relevance [including high-risk clones such as ST11, ST17, and ST512 (38)], making possible to investigate whether these features could be correlated with resistance to defensins. In other words, one could think that higher antibiotic resistance could be related to increased resistance to hBD-3 because of collateral cross effects of certain antibiotic resistance mechanisms. Additionally, it could be argued that the success of epidemic clones could be partially due to increased resistance to host immune resources, as previously described (4). However, none of these possibilities was clearly supported by our results. These circumstances were neither seen for our *A. baumannii* strains [also showing high-level resistance and epidemiological relevance (ST2 epidemic clone)] nor for *E. cloacae* complex ones (some of them also belonging to high-risk clones such as ST78, ST108, and ST114 and displaying high resistance levels) (39).

Our *P. aeruginosa* collection (25), including different profiles of antibiotic resistance and epidemic clones from both acute (ST111, ST175, and ST244) and cystic fibrosis (ST146 and ST274) contexts (40), added a key interesting point to previous studies: investigating whether adaptation to chronic infection could entail consequences for susceptibility to HNP-1 or hBD-3 defensins. In this sense, it has been shown that cystic fibrosis niche adaptation is often correlated with certain collateral virulence attenuated features [e.g., increased complement susceptibility (25)], a circumstance we did not see with hBD-3 activity, but rather the contrary. Future work with higher numbers of strains should be performed to confirm these apparent trends suggesting that chronic adaptation is associated with the selection of increased resistance against the humoral immune resource hBD-3. In any case, this fact would be in accordance with some studies demonstrating that different lipopolysaccharide modifications (e.g., lipid A acylation patterns, aminoarabinose addition, etc.) which are often selected during chronic adaptation may reduce the permeability of Gram-negative envelopes to various antimicrobial peptides (41–43). It would also be in line with the well-known habitual selection for increased antibiotic resistance profiles in long-term cystic fibrosis-adapted *P. aeruginosa* strains (44).

Our data suggest that species is the other factor that seemingly conditions the susceptibility level to the bactericidal power of hBD-3. In this regard, besides the classical conception of lipid II-targeting defensins being more active against Gram-positives because of the absence of outer membrane (6–8), our results indicate that *A. baumannii* and *E. cloacae* complex (this latter species had been never studied in this context before) showed higher susceptibility to hBD-3 than *P. aeruginosa* and *K. pneumoniae*. This is in accordance with data (10) showing that the activity of defensins was highly variable depending on the Gram-negative species considered; with, for instance, *Burkholderia cepacia* complex revealed as being extraordinarily resistant to hBD-3. At any rate, using the breakpoints defined by Sahly et al. (17), all our *E. cloacae* complex isolates (standing out because of their extremely regular behavior, i.e., all showing the same LCs) and all our *A. baumannii* strains would be considered as susceptible (MBCs < 12.5 mg/L), suggesting the interesting potential of this defensin as a therapeutic adjuvant. Moreover, it must be considered that the assays performed here regarding LCs were very strict in terms of estimating bactericidal capacity, since the total eradication of a bacterial population of ca. 10^5^ cells was required in only 2.5 h. Therefore, despite being achieved through dosages in the µM range, this output was reached against many of the isolates studied, demonstrating an antibacterial capacity that is not despicable but rather the contrary for hBD-3. Obviously, future work delving into formulations for topical or inhaled administration, pharmacodynamics, bioavailability, toxicity, resistance to degradation, and other *in vivo* parameters, should be approached in order to confirm this perhaps hBD-3 underrated potential. In fact, several studies searching for artificially modified defensin derivatives with improved properties have been addressed (simplified scaffolds, chimeric/oligomeric variants, peptides synthesized with D-amino acids or with specific amino acid changes to obtain boosted resistance to degradation, encapsulation in silica nanoparticles or impregnation of cotton gauzes to treat infected wounds, etc.) (4, 6–8, 45, 46), but much more knowledge on the topic is still needed.

We previously demonstrated that permeabilization of the external membrane increases the activity of peptidoglycan-lytic immune weapons (lysozyme and PGLYRPs) against *P. aeruginosa*, and that when peptidoglycan recycling is blocked, these aggressions are more productive in terms of bacterial killing. This is logical given that peptidoglycan turnover/recycling is an essential pipeline of new material for the anabolic pathways of cell wall building/repair; thus, if this recycling is blocked, the consequences of any lytic attack should be even more drastic for cell viability (21, 22, 25). Hence, we wanted to determine whether similar effects could be observed with our starring defensins, since although they are not directly lytic on peptidoglycan, they allegedly weaken this essential structure by disabling lipid II function. In other words, we sought to test whether HNP-1/hBD-3 activity could be increased in the presence of an additional permeabilizer (subinhibitory colistin), a peptidoglycan-lytic attack (lysozyme), or in strains in which cell wall recycling is disabled (21, 22, 25). Additionally, we wanted to test the possibility of hBD-3 acting as a permeabilizer and/or posing a peptidoglycan destabilization additional to that caused by meropenem, finally causing a recovery of the effectiveness of this drug against carbapenem-resistant strains. However, none of these combinations gave rise to appreciable improvements in antibacterial activities. From this fact, some points can be made:

1. In relation to the peptidoglycan recycling blockade not causing an increase in hBD-3/HNP-1 bactericidal power, one explanation is that a lytic attack (e.g., lysozyme or PGLYRP2) capable of causing rapid cell death is not the same as disabling the incorporation of new cell wall compounds (lipid II blockade), which could exert its effects in the longer term, i.e., inhibiting growth, which was not seen in our conditions used for bactericidal activity assays. In any case, the peptidoglycan recycling-defective mutants studied here did not show increased susceptibility to the inhibitory capacity of hBD-3 in microdilution assays either (Table 4). Therefore, it seems logical that blocking the incorporation of new peptidoglycan units through defensin activity in recycling-defective mutants had no potentiation effects on their antibacterial capacity as strong as those previously seen when directly degrading peptidoglycan (e.g., lysozyme) in a recycling-defective background (21, 22, 25). However, our results here have been obtained in *P. aeruginosa*, *K. pneumoniae*, and *E. cloacae* peptidoglycan recycling-defective mutants; thus, it remains to be investigated whether *A. baumannii* mutants to be constructed could show increased susceptibility to the lipid II-targeting defensins.
2. Dealing with the combination of defensins plus lysozyme not leading to better bactericidal activity, one can deduce that HNP-1/hBD-3 probably exert a permeabilizing effect that is not efficient enough to enable significant penetration of lysozyme into the periplasm (as, for instance, subinhibitory colistin does in *P. aeruginosa*) (21). In fact, particular mechanisms of membrane permeation have been described for different antimicrobial peptides (toroidal pore, carpet model, etc.), with specifically associated physical-chemical implications (4) that could condition the entrance of high molecular mass molecules such as lysozyme (ca. 14 kDa), thereby not effectively reaching the peptidoglycan target in our conditions. Accordingly, it has been previously reported the lack of synergy between hBD-3 and lysozyme against *E. coli*, which would also support our results (47).
3. Regarding the combination of HNP-1/hBD-3 with subinhibitory colistin not causing any improvement in their bactericidal power, one explanation could be that these defensins may not have a capacity to block lipid II strong enough to significantly kill the studied species. Instead, their anti-Gram-negative activity may rely mainly on permeation, as described for most cationic antimicrobial peptides (4). Thus, an alleged better arrival of the defensin at lipid II thanks to colistin-mediated permeabilization (besides the self-permeabilizing effect exerted by the peptide) would not increase bacterial death. In fact, it has been previously proposed that HNP-1 and hBD-3 may have a relatively low affinity for lipid II in comparison with other compounds (6–8), which supports what our results suggest. Moreover, since subinhibitory colistin has been shown to enable a better arrival of lysozyme to *P. aeruginosa* peptidoglycan (21, 22, 25), and since this enzyme has a molecular weight at least threefold higher than our starring defensins, colistin used here should also improve their entrance into periplasm, again suggesting a poor lipid II-targeting effectiveness.
4. Finally, regarding the meropenem-hBD3 combination, there is evidence supporting that cationic antimicrobial peptides often provide poor outcomes in terms of inhibiting bacterial growth in typical microdilution assays. This is due to different circumstances such as high salt concentration of Müller-Hinton broth, electrostatic sequestration of the peptide by the material of the plates used for microdilution, peptide degradation, and so on (15, 48, 49). Accordingly, the synergies of certain membrane-permeabilizing antimicrobial peptides with classical antibiotics have been reported to be very poor (50). However, a few studies have shown interesting inhibitory capacities for some mammalian/avian defensins against certain Gram-positive and Gram-negative species, while many others have demonstrated considerable synergies of different antimicrobial peptides (although almost never defensins) with conventional antibiotics (51–54). Therefore, to gain knowledge on this somehow controversial field, we wanted to see whether or not hBD-3—selected because of its greater bactericidal capacity in our studies compared to HNP-1—could have a potentiation effect for the relevant β-lactam meropenem. However, our results showing that meropenem MICs did not substantially decrease after combination with hBD-3 (just one dilution in some strains) could also confirm the abovementioned idea concerning the actual capacity of this defensin to effectively disable the lipid II precursor in Gram-negatives (6–8). That is to say, the peptidoglycan-building blockade exerted through hBD-3-mediated lipid II targeting would not be significant enough to substantially increase the peptidoglycan alterations/bacterial death caused by meropenem alone through the typical β-lactam-mediated inhibition of PBPs. On the other hand, regardless of hBD-3 being or not able to significantly block lipid II, our results suggest that an increased arrival of meropenem in the periplasm enabled by the permeabilizing effect of this defensin would still not be enough to sensitize the studied strains against carbapenems (50).

### Conclusions

This is the first study comparatively analyzing the bactericidal power of lipid II-targeting human defensins (HNP-1 and hBD-3) against ESKAPE Gram-negatives using relevant collections of clinical isolates. Our results demonstrate interesting and perhaps neglected levels of hBD-3 bactericidal activity against multidrug resistant *A. baumannii* and *E. cloacae* complex isolates, which is greater than against *K. pneumoniae* and *P. aeruginosa*. Further, our data show an increased resistance of chronically adapted strains of the latter species compared to those from acute infection against the activity of hBD-3. Future work with wider collections is needed to ascertain whether our results are representative of the real scenario for each studied species and to investigate the actual potential of hBD-3 as a therapeutic adjuvant, a possibility enhanced by its low cytotoxicity and notable resistance to physiological salt concentrations (27–31). Meanwhile, we also demonstrate that the peptidoglycan recycling blockade, or combination with lysozyme, β-lactams, or subinhibitory colistin are seemingly not valid strategies to increase the power of HNP-1 or hBD-3. These facts suggest that these defensins may exert their bactericidal effects mostly through permeation of membranes rather than the blockade of lipid II, at least in the Gram-negative species studied.

## MATERIAL AND METHODS

### Strains

Four collections of ESKAPE Gram-negative clinical strains were used in this study. The first, made up of 12 *P. aeruginosa* strains from acute infection (bloodstream) and 12 from cystic fibrosis patients (chronic pulmonary infection), was previously characterized by our group (25). The cystic fibrosis strains were organized in pairs of sequential isogenic isolates from the same patient [early-late, obtained with a difference of at least 3 years; the last two numbers, in italics, of each isolate codification indicate the year of isolation (Table 1)]. Three collections of 10 *E. cloacae* complex, 10 *A. baumanii,* and 12 *K. pneumoniae* strains isolated at University Hospital Son Espases (Palma, Spain) from March 2017 to December 2021 were also included. The strains from these three latter collections—selected because of their high-level resistance (always including carbapenems) and representative lineage relevance—were routinely characterized in the Microbiology Department of the abovementioned hospital: susceptibility profile using a MicroScan WalkAway device and NMDR or NM57 panels (Beckman Coulter) applying EUCAST 2023 (v13.0) clinical breakpoints; E-test (bioMérieux) to determine the minimal inhibitory concentration (MIC) of colistin in selected strains; whole-genome sequencing (WGS) through workflows previously used by our group (55) to determine sequence types (STs) and antibiotic resistance markers; PCR followed by Sanger sequencing (Macrogen) to ascertain the presence of β-lactamase genes typically found in *A. baumannii*; and Pulsed Field Gel Electrophoresis (PFGE) to determine clonal relatedness in *E. cloacae* complex and *K. pneumoniae* strains. The relevant characteristics of all these clinical strains are shown in Tables 1 and 2, together with those of the references *P. aeruginosa* PAO1 and PA14, *K. pneumoniae* Kp52.145 and ATCC 700721, *E. cloacae* ATCC 13047, and *A. baumannii* ATCC 19606, also used in this work.

Additionally, several mutants KO in different targets essential for peptidoglycan recycling, previously constructed following standard procedures (56–58), were used to determine whether a higher activity of HNP-1/hBD-3 against them compared to originative strains could be appreciable. For *P. aeruginosa,* the PAO1- and PA14-derived mutants defective in AmpG permease (PAΔAG and PA14ΔAG), the N-acetyl-glucosaminidase NagZ (PAΔnZ and PA14ΔnZ) and the three amidase homologues AmpD, AmpDh2, and AmpDh3 (PAΔDDh2Dh3 and PA14ΔDDh2Dh3) were included (21, 54–56). Regarding the other species, only the previously constructed *ampG* KO mutants Kp52.145RΔAG and MGH 78578ΔAG (*K. pneumoniae*) and ATCC 13047ΔAG (*E. cloacae*) were used (59, 60).

### Antibacterial activity

Previously described protocols (11, 17, 61) with minor modifications were followed to assess the bactericidal activity of purified mature L-amino acids HNP-1 (ACYCRIPACIAGERRYGTCIYQGRLWAFCC) and hBD-3 (GIINTLQKYYCRVRGGRCAVLSCLPKEEQIGKCSTRGRKCCRRKK) (Eurogentec) against the abovementioned strains. Exponentially growing bacteria were washed with 10 mM sodium phosphate buffer (pH 7.4) and diluted in the same buffer to 10^6^ colony-forming units (CFU)/mL. A final volume of 100 µL was used in 96-well plates to determine the activity of HNP-1 and hBD-3 (dissolved in the same buffer). After incubation with serial concentrations of these defensins for 2.5 h at 37°C, contents of wells were cultured on LB plates, which were incubated for 18 h at 37°C. The lowest concentrations of HNP-1 or hBD-3 at which there were no CFUs left, were considered the lethal concentrations (LCs), whereas those required to kill ≥99% of bacterial inoculums were defined as the minimum bactericidal concentrations (MBCs) (11, 17, 61). When indicated, human lysozyme (Sigma-Aldrich) to reach a fixed physiological concentration of 25 mg/L, and colistin sulfate (Sigma-Aldrich) to reach a fixed subinhibitory concentration of 0.025 mg/L (25) were added to each defensin treatment, maintaining the same final volume/well. When specified, incubations with HNP-1 or hBD-3 were extended to 18 h at 37°C, increasing the volume/well to 200 µL to minimize evaporation issues.

In order to analyze whether a combination of hBD-3 with β-lactams could improve the activity of these latter, Müller-Hinton broth microdilutions to determine the MICs of hBD-3, meropenem (Aurovitas), and combined treatment were performed following previously described protocols (15) against selected strains. In the combined treatment, hBD-3 was added in all instances in sub-lethal concentrations: 1/4 of the LC previously obtained for each strain.

In all cases, the *Micrococcus lysodeikticus* ATCC 4698 strain was used as a Gram-positive control for the activity of defensins (15). All the displayed results of LCs, MBCs, and MICs against the different species/strains are the median values obtained from three independent experiments.

## Data Availability

The data sets generated during the current study are virtually displayed in full in the manuscript (Tables 1 to 4), but also available from the corresponding author on reasonable request.
